# Supplementary material for: The fibronectin synergy site re-enforces cell adhesion and mediates a crosstalk between integrin classes
Source: eLife. 2017 Jan 16;6:e22264. doi: 10.7554/eLife.22264 (PMC5279944; doi:10.7554/eLife.22264)
Supplement: Table 1—source data 1. — DOI: http://dx.doi.org/10.7554/eLife.22264.017 [file elife-22264-table1-data1.docx]

**Table 1-source data 1.** Progeny of *Fn1^syn/syn^*;*Itgb3^+/-^* x *Fn1^syn/syn^*;*Itgb3^+/-^* crosses

| Age | Number | *Fn1^syn/syn^Itgb3^+/-^* | *Fn1^syn/syn^Itgb3^+/+^* | *Fn1^syn/syn^Itgb3^-/-^* |
| --- | --- | --- | --- | --- |
| E11.5 | 13 | 4  (31%) | 6  (46%) | 3  (23%) |
| E14.5 | 31 | 7  (22.6%) | 14  (45.2%) | 10  (32.2%) |
| E15.5 | 9 | 3  (33%) | 3  (33%) | 3  (33%) |
| E16.5 | 31 | 8  (26%) | 19  (61%) | 4  (13%) |
| P 21 | 90 | 25  (27.7%) | 64  (71%) | 1  (1.1%) |
| Mendelian Distribution | 100 | 25% | 50% | 25% |
